# Individual differences in response conflict adaptations

**DOI:** 10.3389/fpsyg.2013.00947

**Published:** 2013-12-18

**Authors:** Doris Keye, Oliver Wilhelm, Klaus Oberauer, Birgit Stürmer

**Affiliations:** ^1^German Aerospace Center, Institute of Aerospace MedicineHamburg, Germany; ^2^Institute of Psychology and Education, University UlmUlm, Germany; ^3^Department of Psychology, University of ZurichZurich, Switzerland; ^4^International Psychoanalytic UniversityBerlin, Germany

**Keywords:** conflict-monitoring, conflict, working-memory capacity, executive attention, frequency of conflict

## Abstract

Conflict-monitoring theory argues for a general cognitive mechanism that monitors for conflicts in information-processing. If that mechanism detects conflict, it engages cognitive control to resolve it. A slow-down in response to incongruent trials (conflict effect), and a modulation of the conflict effect by the congruence of the preceding trial (Gratton or context effect) have been taken as indicators of such a monitoring system. The present study (*N* = 157) investigated individual differences in the conflict and the context effect in a horizontal and a vertical Simon task, and their correlation with working memory capacity (WMC). Strength of conflict was varied by proportion of congruent trials. Coherent factors could be formed representing individual differences in speeded performance, conflict adaptation, and context adaptation. Conflict and context factors were not associated with each other. Contrary to theories assuming a close relation between working memory and cognitive control, WMC showed no relation with any factors representing adaptation to conflict.

## Introduction

Sometimes we have to make choices that go against learned habits or natural response tendencies, such as moving a lever to the left to make a right turn. These situations generate response conflicts. Cognitive control can be recruited to assist conflict resolution and to choose an answer appropriate to the person's current goals, overcoming strong but wrong response tendencies. Some researchers have argued that the recruitment of cognitive control relies on a system that continuously monitors for conflicts in information processing (Botvinick et al., 2001; Kerns et al., 2004). Such a cognitive system is argued to (1) rely on the monitoring of conflicts in information processing, (2) increase control when it is required by high degrees of conflict, and (3) trigger learning processes based on the experiences from conflict (Botvinick, 2007).

In experimental response-conflict paradigms such as the Simon task, the Stroop task, or the Eriksen Flanker task, people carry out a choice task on stimuli that have a relevant feature determining the required response, and at least one irrelevant feature that is also associated with one of the response alternatives. In these paradigms an irrelevant stimulus feature can facilitate the correct response by activating the same response as the relevant stimulus feature (congruent trial) or elicit a response tendency opposite to the correct response (incongruent trial), thus creating response conflict. For instance, in the Simon task (Simon, 1990), people make manual choice responses on the basis of a non-spatial stimulus feature (e.g., form). Response keys are arranged on a spatial dimension (e.g., one left, one right), and the stimulus location varies randomly along the same spatial dimension (i.e., they are displayed left or right of the midline). Thus, the task- irrelevant dimension of stimulus location overlaps with the spatial arrangement of the response. The Simon effect refers to the finding that responses are usually faster and less error-prone in case the task-irrelevant stimulus location corresponds to the location of the correct response, whereas responses are slowed down in case stimulus location and response position mismatch. According to dual-route models this performance impairment is related to conflict between two mutually exclusive response tendencies (Kornblum et al., 1990; De Jong et al., 1994). Dual-route models incorporate an indirect response preparation route that is characterized by top-down controlled response selection relying on task instructions, and a direct route that automatically primes responses on the basis of overlapping stimulus and response characteristics. In congruent trials the spatial response selected by indirect route processing is already activated via direct-route priming. In incongruent trials the direct route primes a response different from that selected by the indirect route, resulting in a conflict between response tendencies that has to be resolved before the correct response can be executed, thus prolonging RT. Conflict slow-down effects have been established and replicated for the Simon task (Simon, 1990), the Eriksen Flanker task (Eriksen and Hoffman, 1973; Eriksen and Schultz, 1979), and the Stroop task (MacLeod, 1991).

In the conflict monitoring theory (Botvinick et al., 2001, 2004) it is argued that the level of conflict between two (or more) response alternatives is continuously measured. In the case of sufficient conflict the monitoring system initiates cognitive control processes that strengthen the indirect route relative to the direct route, thereby biasing conflict resolution on the present trial toward the instruction-appropriate response. Such a conflict monitoring system ensures fluent and efficient behavior by calling on cognitive control only when required—thus saving cognitive resources when they are not required, and enabling behavior to benefit from environmental input as long as it is congruent with the goal-appropriate response (Botvinick, 2007). Conflict monitoring theory was supported by well replicated experimental effects of the context dependency of conflict (Gratton et al., 1992; Stürmer et al., 2002; Kerns et al., 2004; Ullsperger et al., 2005; Freitas et al., 2007). According to the conflict monitoring theory, an incongruent trial leads to an activation of cognitive control that carries over into the next trial. Therefore, an incongruent trial *n* − 1 results in a relative suppression of the influence of irrelevant stimulus features on trial *n*, compared to a congruent trial *n* − 1. As a consequence, incongruent trials *n* following incongruent trials *n* − 1 (a sequence we denote as iI) are faster than those following congruent trials *n* − 1 (cI) because of reduced conflict from the irrelevant feature in the first case. Conversely, congruent trials *n* following an incongruent trial *n* − 1 (iC) are slower than congruent trials *n* following congruent trials *n* − 1 (cC) because the iC trials benefit less from priming of the correct response by the irrelevant feature.

The explanation of the context effect by conflict monitoring was challenged by Mayr et al. (2003) as well as Hommel (2004), both showing that repetition priming could explain the modulation in the conflict effect without any need for a construct like cognitive control. However, several studies have shown that response priming alone was insufficient to explain the context effect (Stürmer et al., 2002; Ullsperger et al., 2005; Notebaert et al., 2006; Freitas et al., 2007; Keye et al., 2009).

### Individual differences and cognitive control

Taking an individual-differences perspective, Kane and Engle (2003) argued that the ability to inhibit irrelevant information reflects working memory capacity (WMC). Kane et al. (2007) interpret WMC as reflecting the ability to control attention especially in face of interfering information, an ability they call executive attention (EA). According to this theory people with low WMC are impaired in tasks calling for EA compared with people with high WMC. More specifically, individuals low in WMC are more strongly affected by conditions of interference than those high in WMC. This prediction has been confirmed experimentally in extreme group designs for the Stroop task (Kane and Engle, 2003), the antisaccade task (Kane et al., 2001), the flanker task (Heitz and Engle, 2007) and the dichotic listening task (Conway et al., 2001), among others.

Similarly, Lavie and colleagues proposed their *load theory* of attention in which WMC reflects the ability to maintain the current task-goal and focus attention on the relevant stimuli, at the same time blocking interference from irrelevant stimuli (Lavie et al., 2004; Lavie and De Fockert, 2005). High demands on WMC impair the ability to actively maintain the relevant information in the scope of attention (Lavie et al., 2004). Considering individual differences, this theory implies that people low in WMC should be more susceptible to interference from irrelevant stimuli than people high in WMC.

EA theory, load theory, and conflict-monitoring theory assume a central system for cognitive control in response to conflict. Taking theories together, we should expect individual differences in the two signature effects supporting conflict-monitoring theory, the conflict slow-down and the context effect. These individual differences should be related to measures of WMC. Using confirmatory measurement models, we aim to replicate and extend previous findings by Keye et al. (2009). Keye et al. (2009) established task-specific latent factors for conflict and context slow-down that, however, were only weakly correlated across different conflict task paradigms, and only weakly associated with WMC, contrary to the expectations from EA and load theory.

Borgmann et al. (2007) have raised concerns about the reliability of measures of cognitive processes in conflict tasks like the Simon task. They could show that the reliability of the conflict effect in a Simon task increased with the relative frequency of the congruent trials. Borgmann et al. (2007) interpreted this result in line with EA theory (Kane and Engle, 2003; Engle and Kane, 2004). Conflict in incongruent trials grows stronger when congruent trials are more frequent because there are fewer trials that trigger an increase of cognitive control. EA theory assumes that when incongruent trials are rare, people rely more strongly on the direct route, and the instructed task set becomes less available, leading to occasional “goal neglect” (Kane and Engle, 2003). Therefore, cognitive control demands in incongruent trials are supposedly higher when incongruent trials are relatively rare. Kane and Engle (2003) argued, WMC should correlate with the efficiency of conflict resolution particularly when incongruent trials are rare.

More encouraging results for the assumption that WMC and conflict monitoring are closely related comes from brain imaging studies that have linked both demands on WMC and demands on cognitive control to frontal cortex areas. Participants with high WMC have higher anterior cingulate cortex (ACC) activity when working on a working-memory task (Osaka et al., 2003). High ACC activity has been interpreted as reflecting a more efficient cognitive control system. The ACC has been repeatedly linked to conflict monitoring and cognitive control (for reviews see Botvinick, 2007; Carter and van Veen, 2007). In line with the idea of stronger conflict resulting in higher demands on cognitive control, infrequent responses or unpredictable events have been found to go along with heightened ACC activity (Braver et al., 2001; Nieuwenhuis et al., 2003; Hahn et al., 2007).

### Research questions

In an attempt to integrate the ideas of the conflict monitoring theory, the theory of EA, and load theory, we pursued the following three goals. First, we wanted to investigate individual differences in conflict slow-down and its context dependency. If (a) a central mechanism initiates control processes as suggested by conflict monitoring theory, and (b) there are individual differences in the efficiency of this central mechanism, we would expect positive correlations among individual measures of the size of the conflict and the context effect across different tasks, because they are all indicators of the strength of a general cognitive control mechanism. Second, if WMC reflects the ability to deal successfully with interference in information processing, as assumed by the EA and load theory, measures of WMC should be correlated with conflict effects. Third, if frequency manipulations affect cognitive control demands, people with lower WMC should be particularly impaired when incongruent trials are less frequent than congruent trials (Kane and Engle, 2003).

## Methods

### Participants

One hundred and fifty seven persons participated in this study. They were recruited through a university e-mail list server. Twenty persons were dropped from all further analysis because they were not sufficiently compliant displaying unreasonably long RT or very low accuracy rates in at least one of the conditions or did not complete all tasks. All analyses are based on the data from the remaining 137 participants (40 males). Participants had a mean age of 24.6 years (SD 3.9 years) the youngest being 18 years and the oldest being 39 years of age.

### Measures

Apart from a demographic background questionnaire all data were collected on identical computers with 17 inch monitors. The resolution was set to 1024 × 768 pixels with a refresh rate of 85 Hz. Inquisit 2.0© was used to run all computerized tasks.

A horizontal and a vertical version of the Simon task paradigm were administered. Diamonds and squares were used as stimuli for the vertical and for the horizontal Simon task. Shapes appeared above or below a centered fixation cross (vertical version) or to the left or right of a centered fixation cross (horizontal version). Both shapes measured 30 × 30 mm and appeared in 85 mm distance from the center of the screen. Shapes remained on the screen until the participants responded. Response devices were vertically or horizontally arranged keys, for the vertical and for the horizontal version of the Simon task, respectively. Participants responded by pressing one of two keys vertically or horizontally arranged on a custom built keyboard using their index fingers of both hands. The upper key for the vertical task was labeled with an upward pointing arrow, and the lower key with a downward pointing arrow. Regarding the horizontal task, the left-sided key was labeled with a left pointing arrow and the right-sided key was labeled with a right pointing arrow. The diamond shape was assigned to the upper or left key, and the square to the lower or right key. Thus for the vertical Simon task, a diamond in the upper half of the screen, or a square in the lower half, were congruent trials, whereas a diamond in the lower half and a square in the upper half were incongruent stimuli.

The trial settings were identical for both versions of the Simon task. Each trial started with a centered fixation cross, followed after 500 ms by the target stimulus. The target stimulus remained on screen until the participant responded with a valid key press. The interval between a response and the next fixation cross was set to 1000 ms.

Block settings depended on the proportion of congruent trials. The proportion of congruent trials was varied in the horizontal task between 25, 50, and 75%. This led to three different horizontal Simon tasks that were administered in separate blocks. The vertical Simon task was only administered with a congruency rate of 50% congruent stimuli. Block settings for the 50% congruency tasks were eight blocks with 41 trials, the first trial being a warm-up trial that was discarded in all subsequent analysis. To ensure a sufficient number of congruent or incongruent trials per condition, and to allow for tests of sequence modulation, 16 blocks each were administered for the 25% and the 75% congruency conditions with 41 trials per block. Participants received one practice block except when they performed the vertical and one of the horizontal Simon tasks for the first time—then participants started with two practice blocks. Practice blocks only had 15 trials because of the simplicity of the task. Feedback was given after every block indicating average performance in milliseconds and the percentage of correct responses in the block that was just completed.

The experimental design for the horizontal task was a 2 (current trial: congruent vs. incongruent; indicated by a capital C or I) by 2 (previous trial: congruent vs. incongruent; indicated with a lower-case c or i) by 2 (stimulus sequence: identical vs. non-identical stimuli) by 3 (congruent trial frequency: 25, 50, 75%) within subject-factor design. The sequence of the three horizontal Simon tasks differing in frequency of congruent trials was randomly varied between subjects by using the six possible sequences of the three conditions equally often. The design for the vertical task was the same, except for the frequency variable which was held constant at 50%.

WMC was measured with three tasks: memory updating, counting span, and rotation span. In the memory-updating task, participants had to memorize numbers and their positions in a grid, and update the numbers by applying simple arithmetical operations to them (Oberauer et al., 2000). In the counting span task (Case et al., 1982) participants counted sets of blue circles and memorized each count, while being distracted by blue squares and green circles also present on the displays. Besides memorizing the count participants additionally had to decide whether each count was odd or even by pressing one of two keys. After a series of 2–6 displays the counts had to be reproduced in their correct order (Wilhelm and Oberauer, 2006). In the rotation span task (Shah and Miyake, 1996) participants had to memorize a sequence of arrows that vary in length and pointing direction while between the presentations of every two arrows participants had to decide whether or not rotated letters were mirror-reversed by pressing one of two keys.

The experiment started with a demographic questionnaire and tasks were administered in the following sequence: vertical Simon task, rotation span task, horizontal Simon task (first congruency manipulation), counting span task, horizontal Simon task (second congruency manipulation), memory updating task, horizontal Simon task (third congruency manipulation). Participants received a 5 min break after the counting span task. In order to control for sequence effects due to the manipulation of the proportion of congruent trials, six different permutations of sequence order were administered. The sequence manipulation affected only the horizontal Simon tasks; all order of all other tasks in the experiment was fixed.

### Data analyses

RT of wrong responses were excluded from analyses of the Simon task data. A trimming procedure was applied to eliminate unreasonably short and long RT. All responses with RT lower than 100 ms were set to missing values. An upper boundary was defined by the individual RT mean plus three intraindividual standard deviations. All RT above this limit were set to missing values. The trimming procedure was applied iteratively with newly computed means and standard deviations until no RT were excluded in two subsequent runs.

Partial credit scoring procedure was used for the WMC tasks (Conway et al., 2005). In the counting span and rotation span tasks participants had to be correct in at least 75 percent of the secondary tasks (odd-even decision and mirror-reversal decision, respectively). This limit ensured that participants had worked on the secondary task sufficiently to affect performance on the primary task. This was not achieved by two participants for the counting span task and 10 participants for the rotation span task, these data points were set to missing values. Missing values were substituted by Missing Value Analysis (SPSS 12.0) using the estimation maximization (EM) procedure after Little's Missing Completely at Random test (MCAR, Little, 1988) which was run to ensure that the substitution was justified. MCAR-test statistics for the working memory tasks was not significant (χ^2^ = 8, *df* = 4, *p* = 0.10). Therefore, in order to exhaust the available data the missing values were imputed with the EM procedure.

Individual differences in experimental effects for RT and accuracies were analyzed with structural equation modeling (s.e.m) using AMOS 7.0© with Maximum-Likelihood (ML) estimation. Reported results for individual differences will focus on RT data, though accuracies were analyzed as well. Supplemental material provides the s.e.m results of accuracy data, which support the same conclusions as those of the RT data.

**Figure 1:**
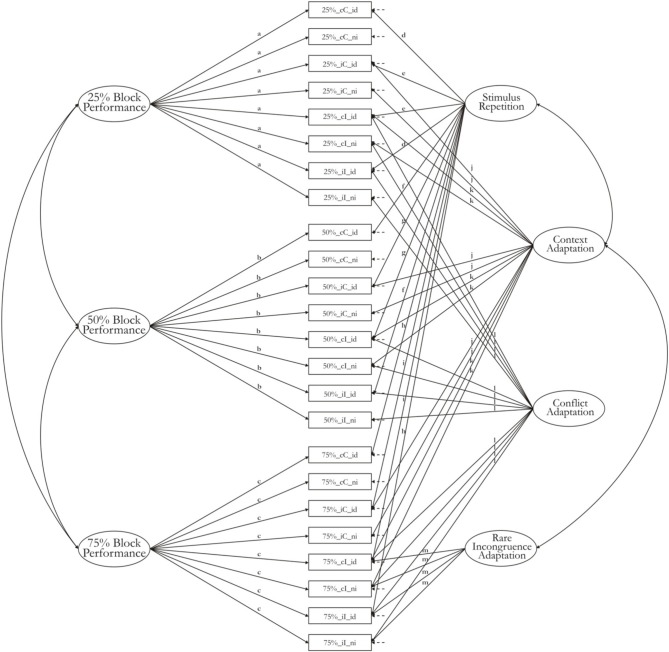
**Measurement model with pattern of constraints for the horizontal Simon tasks**.

We used a model structure proposed for experimental designs by Oberauer et al. (2005). The model is applied to the means of all indicator variables as well as their variance-covariance matrix, thereby capturing the experimental effects on both means and individual differences simultaneously. General task performance factors with loadings from all indicators of the task block capture variance in those cognitive processes that are common to all experimental conditions (i.e., the baseline of the experimental design). Nested factors on which only a subset of indicators had free loadings served to represent experimental effects. For instance, all variables reflecting RT on incongruent trials had free loadings on the Conflict-Adaptation factor, whereas all variables reflecting RT on congruent trials had their loadings on this factor fixed to zero. In this way, the mean of the Conflict-Adaptation factor reflects the mean conflict effect, and variance in the Conflict-Adaptation factor reflects individual differences in the size of the conflict effect (i.e., the RT difference between congruent and incongruent trials). Loadings were constrained to be the same for groups of indicators reflecting same experimental conditions. The pattern of constraints on loadings reflects the assumptions about additive and non-additive effects of experimental variables. For instance, a fully additive model would constrain the loadings of all indicators on a factor to be the same, regardless of what other factors the indicators also loaded on. This set of constraints reflects the assumption that the effect of one factor on an indicator variable is independent of the effect of other factors on that indicator variable. In practice, the assumption of pure additivity rarely holds (for an exception see Ecker et al., 2010). We chose a set of constraints that reflects a number of interactions—for instance, the loadings of the context factors were assumed to differ with the congruency of the current trial, and therefore, we allowed two different values of loadings on the Context-Adaptation factor, one for congruent and one for incongruent trials. Figure 1 presents the structure of the initial model together with constraints on the loadings.

Adequacy of models was judged by inferential and descriptive fit statistics including the χ^2^-test statistic, the Comparative-Fit-Index (CFI) and the Standardized-Root-Mean-Square-Residual (SRMR) (Hu and Bentler, 1999). Competing nested models were compared with the help of likelihood ratio tests. Factor reliability is provided by McDonald's omega which indicates the variance a latent factor captures considering the total variance of a scale (McDonald, 1999). For our purposes this means how much variance the factor captures from its respective indicators.

## Results

Descriptive statistics for all tasks are summarized in Table 1. For ease of presentation, analyses are divided into two sections. First we present the analysis for the horizontal Simon tasks. In this section the effect of congruency frequency manipulations will be focused. Second, the horizontal Simon task with 50% congruency will be compared with the vertical Simon task with 50% congruency. In this section the effects of spatial orientation of the task will be the primary focus.

**Table 1:** **Descriptive Statistics for all Simon Tasks**.

	**RT**		**Accuracy**	
	**Mean (id/ni)**	***SD* (id/ni)**	**Mean (id/ni)**	***SD* (id/ni)**
**HORIZONTAL SIMON TASK 25% CONGRUENCY**				
cC	393/404	58/53	0.98/0.97	0.04/0.05
iC	417/449	50/61	0.94/0.91	0.05/0.06
cI	429/449	51/51	0.93/0.92	0.05/0.06
iI	403/419	46/52	0.98/0.97	0.02/0.03
**HORIZONTAL SIMON TASK 50% CONGRUENCY**				
cC	387/402	45/51	0.98/0.98	0.03/0.03
iC	410/443	58/51	0.96/0.94	0.04/0.05
cI	436/455	51/49	0.93/0.91	0.06/0.07
iI	404/432	47/50	0.98/0.97	0.03/0.04
**HORIZONTAL SIMON TASK 75% CONGRUENCY**				
cC	377/386	42/44	0.99/0.99	0.01/0.01
iC	402/442	49/52	0.97/0.95	0.03/0.04
cI	451/467	50/51	0.85/0.87	0.08/0.08
iI	413/443	47/53	0.98/0.95	0.03/0.06
**VERTICAL SIMON TASK 50% CONGRUENCY**				
cC	396/404	44/49	0.99/0.99	0.02/0.02
iC	432/455	53/59	0.98/0.96	0.03/0.04
cI	465/480	57/61	0.93/0.89	0.06/0.08
iI	436/456	51/60	0.98/0.97	0.03/0.04

cC, congruent trial preceded by a congruent trial; iI, incongruent trial preceded by an incongruent trial; iC, congruent trial preceded by an incongruent; cI, incongruent trial preceded by a congruent trial; id, identical target stimulus in the preceding trial; ni, different target stimulus in the preceding trial.

### Horizontal simon tasks: 25 vs. 50 vs. 75% congruency

Summarizing experimental effects, we replicated the experimental findings from previous studies: The conflict effect was reflected in slower RT and more errors on incongruent than on congruent trials. The context effect was reflected in an interaction of current-trial with previous-trial congruency. The conflict effect was modulated by relative frequency of congruent trials such that when incongruent trials were rare, the conflict effect became stronger, as expected on the assumption that a high proportion of congruent trials lures the cognitive system into a state of relaxed control in which the irrelevant stimulus feature (i.e., spatial location of the stimulus) is allowed a large influence on response selection. We statistically confirmed these mean differences in Two Ways, through ANOVA (detailed ANOVA tables are provided in the appendix) and by including parameters for factor means in the structural equation models (to be described below). In order to control for possible effects of stimulus repetition^1^ an additional ANOVA was performed excluding all trials in which the current relevant stimulus dimension repeated the one from the preceding trial. Results for RT and accuracy still showed a strong two-way interaction between current-trial congruency and preceding-trial congruency, indicative of the context effect, η^2^ = 0.32 [*F*_(1, 136)_ = 6.3, *p* < 0.001] for RT, and η^2^ = 0.26 [*F*_(1, 136)_ = 3.4, *p* < 0.001] for accuracy data.

Individual differences in RT were analyzed with confirmatory factor analysis. Indicator variables were mean RT in each design cell of experimental design. We started with modeling only the variance-covariance structure with unconstrained loadings of indicators on factors. Manifest variables were individual's mean RTs in each cell of the design, computed separately for each block (25, 50, or 75% congruent trials).

General speed on the experimental task was represented by factors on which RTs from all design cells had free loadings. To account for subtle differences between blocks, we had to assume three separate but highly correlated general-speed factors, one for each block. A nested factor for conflict adaptation was specified on which only RTs from incongruent trials had free loadings. This factor reflects individual differences in conflict adaptation, that is, in the degree to which RT on incongruent trials were slower than RT on congruent trials. A nested factor for stimulus repetition, on which all indicators with identical stimuli on the preceding trials had free loadings, represents individual differences in the acceleration of responses due to identical stimulus predecessors. The context adaptation factor, which had loadings from all indicators reflecting iC and cI trial sequences, reflects individual differences in the size of the context effect. Additionally, a rare-incongruent factor with loadings from incongruent trials from the Simon task block with 75% congruent trials, and a rare-congruent factor with loadings from all congruent trials in the block with 25% congruent trials, were included in the model.

This first model (model 1-1) fit the data well (χ^2^ = 4.7, *df* = 205, *p* < 0.01, CFI = 0.96, SRMR = 0.04). However, insignificant loadings for the rare-congruence factor supported its deletion from the model. The remaining rare-incongruent factor reflects individual differences in the degree to which the conflict slow-down is exaggerated in blocks with few incongruent trials, compared to the baseline with 50% incongruent trials. The fit of the resulting model 1-1b was not significantly worse than that of model 1-1 (Δχ^2^ = 5, Δ*df* = 4; χ^2^ = 427, *df* = 205, *p* < 0.01, CFI = 0.96, SRMR = 0.04), and was therefore accepted as the model with unconstrained loadings. Most variance was captured by the general block performance factors compared to the loadings of the nested factors. Even though loadings were considerably lower for nested factors, especially the context adaptation factor, factor loadings were significant, and dropping any of the nested factors resulted in a significant decrease in model fit. We, therefore, conclude that the nested factors represent systematic variance. The fact that loadings on the nested factors were smaller than loadings on the general factors implies that the influence of individual differences in cognitive control is relatively small, compared to the influence of other sources of individual differences, such as general processing efficiency.

In model 1-2 constraints were introduced into model 1-1b to fix loadings to be equal across different indicators from the same experimental condition. The pattern of constraints, summarized in Figure 1, reflects a model with main effects and two-way interactions between experimental effects. That is, loadings of all indicator variables reflecting the same combination of values on two interacting experimental variables were constrained to be the same. For instance, the two main effects of current-trial congruency and preceding-trial congruency, together with their two-way interaction, is captured by a pattern of loadings that allows different means and covariances between but not within the four design cells relevant to this interaction: cC, iC, cI, and iI. The model therefore assigns different loadings to cI and iC variables on the context-adaptation factor. The predicted values for the four cells are now (ignoring the contributions of the repetition and the rare-incongruence factor):

$$\begin{array}{l} cC=\text{baseline}\\ iC=\text{baseline}+j\times \text{context}\\ cI=\text{baseline}+k\times \text{context}+l\times \text{conflict}\\ iI=\text{baseline}+l\times \text{conflict}, \end{array}$$

with baseline referring to the relevant baseline factor for the block (25, 50, or 75% incongruent trials). The constrained model 1-2 fit the data worse than model 1-1b but was still acceptable (χ^2^ = 580, *df* = 258, *p* < 0.01, CFI = 0.95, SRMR = 0.04).

Model 1-3 had the same structure as model 1-2 but included an estimation of the factor means. In this model the intercepts of all indicators were fixed to zero. The main implication of this constraint is that the indicator means (i.e., RT means of the experimental design cells) are predicted from the factor means and the (constrained) loadings, and any residual in the indicator means contributes to the misfit of the model. With this model we attempt to capture the effects of the experimental manipulations on means and on the covariance structure simultaneously. As expected, the fit of model 1-3 dropped relative to the less constrained model 1-2 (χ^2^ = 910, *df* = 275, *p* < 0.01, CFI = 0.90, SRMR = 0.04). Inspection of the estimations for the indicator means displayed exceptionally high residuals for two indicators; these were the iC-non-repetition indicators from the 25% and from the 75% horizontal Simon task blocks. Model 1-3 was modified into model 1-3b by setting the means of the corresponding errors free to allow for this deviation from the imposed structure. The means of the residuals were estimated at 17 ms for the iC-non-identical indicator from the 25% Simon task, and 23 ms for the iC-non-identical indicator from the 75% Simon task. The model fit was improved to χ^2^ = 746, *df* = 273, *p* < 0.01, CFI = 0.92, SRMR = 0.04. Given the strict constraints on model 1-3b, which is punished for misspecifications of variance-covariance structure as well as deviations from indicator means, this fit can still be deemed acceptable.

**Figure 2:**
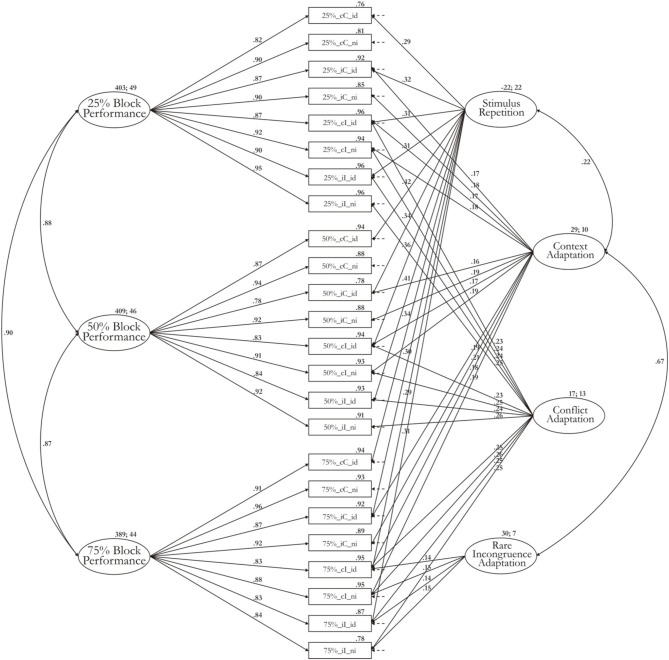
**Measurement model 1-3b including estimates for factor means and factor variance**.

Model 1-3b is illustrated in Figure 2. Table 2 provides the estimated McDonald's ω for each factor. The Appendix provides additionally the unstandardized regression weights with which the effect of each factor mean on the particular RT indicator can be reproduced. Most variance is captured by the three general performance factors, which also display the highest factor means: 403 ms (sd: 49 ms) for the block factor from the 25% Simon task, 409 ms (sd: 46 ms) for the block factor from the 50% Simon task, and 389 ms (sd: 44 ms) for the block factor from the 75% Simon task. The stimulus-repetition factor has a negative factor mean (mean: −22 ms) reflecting the acceleration of responses in trials with repeated stimuli. The standard deviation of this factor is as high as the factor mean (sd: 22 ms) implying that some participants show high repetition effects whereas others show a zero or even negative repetition effect. This result as well as McDonald's ω indicate that there are substantial individual differences in a latent stimulus-repetition factor. The mean of the conflict factor (mean: 17 ms; SD: 13 ms) reflects the slow-down shared by all indicators reflecting trials high in conflict (incongruent trials) compared to trials low in conflict (congruent trials). Similar to the repetition factor, the ratio of factor mean to standard deviation implies that considerable individual differences are associated with this effect. The mean of the rare-incongruence factor is estimated at 30 ms (SD: 7 ms). Less individual differences are associated with the rare-incongruent-trial condition, compared to the conflict-adaptation effect also reflected in a very low value for ω. At the same time, the mean effect is higher than that of the conflict-adaptation effect. This latent factor for conflict adaptation is, hence, mostly necessary to explain significant differences in means rather than variance-covariance structure. Finally, the context factor reflects a slow-down of 29 ms (SD: 10 ms) on trials where the current congruency condition mismatches that on the preceding trial (i.e., iC and cI trials). Individual differences for this factor are weaker in comparison to other factors but are still meaningful considering the captured variance for this factor.

**Table 2:** **McDonald's ω and correlations of RT from the Contrast between the Horizontal Simon Tasks**.

	**RT Data**	
	**McDonald's ω from model (1–3b)**	**Correlations of WM and latent factors from model (1–4)**
RT Performance 25% congruence frequency	0.969	−0.44^**^; Δχ^2^ = 17, Δ*df* = 1
RT Performance 50% congruence frequency	0.964	−0.34^**^; Δχ^2^ = 11, Δ*df* = 1
RT Performance 75% congruence frequency	0.965	−0.36^**^; Δχ^2^ = 12, Δ*df* = 1
Stimulus repetition	0.600	−0.08; Δχ^2^ = 1, Δ*df* = 1
Conflict adaptation	0.436	−0.02; Δχ^2^ = 0, Δ*df* = 1
Context adaptation	0.291	0.04; Δχ^2^ = 0, Δ*df* = 1
Rare incongruence adaptation	0.079	−0.21; Δχ^2^ = 2, Δ*df* = 1

Significance of correlations is indicated by ^*^ for p < 0.05 and

** for p < 0.01.

Correlations between latent factors were estimated based on model 1-3b. The three general block factors were strongly correlated with each other. The context factor was positively correlated with the rare incongruence factor (*r* = 0.67, Δχ^2^ = 13, Δ*df* = 1). Participants who displayed higher context adaptation were also more affected by manipulations of the proportion of incongruent trials. The association between the context and the stimulus repetition factor was weak but still passed significance testing (*r* = 0.22, Δχ^2^ = 4, Δ*df* = 1). The conflict and the context factor were not correlated significantly.

For the purpose of estimating correlations, factor means were neglected. Therefore, to test hypotheses about associations with external criteria, WMC was introduced into model 1-2.rather than 1-3b. The WMC factor had loadings from rotation span (factor loading.79), memory updating (factor loading.70), and counting span (factor loading.50) and was allowed to correlate with all factors in model 1-2. There were no constraints on the WMC factor loadings. The fit of the resulting model 1-4 was acceptable (χ^2^ = 673, *df* = 324, *p* < 0.01, CFI = 0.94, SRMR = 0.05). Correlations of WMC with latent factors are summarized in Table 3. WMC showed a significant negative correlation with the general block performance factors. Participants high in WMC tended to respond generally faster than people low in WMC. WMC showed no significant associations with any other of the RT factors.

**Table 3:**
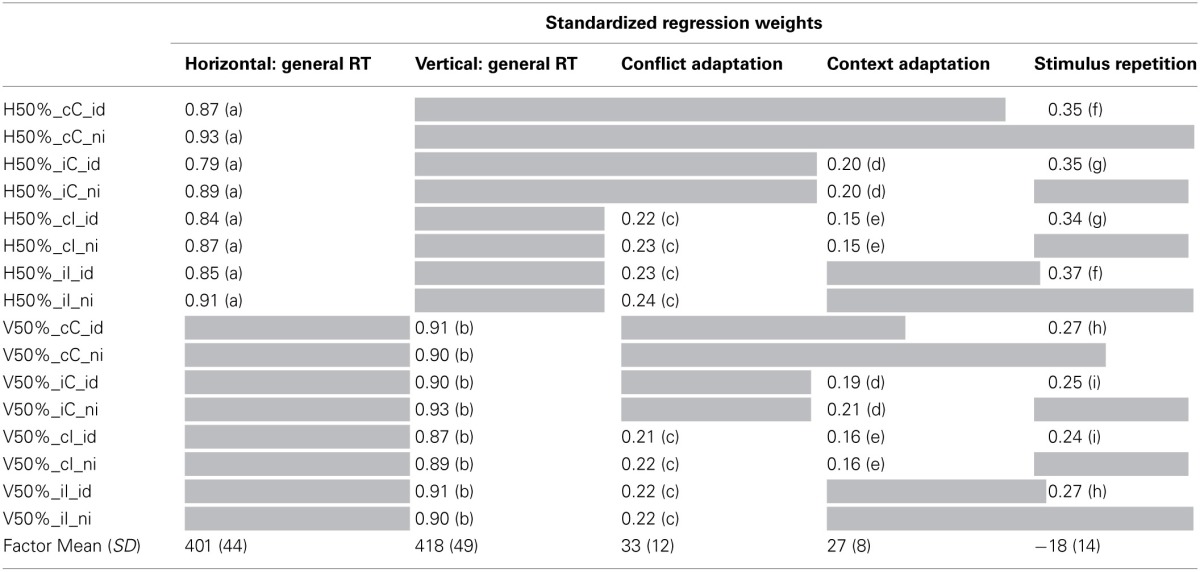
**Factor Means, *SD* and Factor Loadings from Model 2-2**.

Letters in brackets indicate the constraints set on factor loadings. Gray-shaded arrays indicated fixation to zero.

### Vertical 50% congruency vs. horizontal 50% congruency

An ANOVA for RT contrasting the horizontal and the vertical Simon task with 50% congruent trials (see Tables in appendix) revealed that the spatial orientation had only a marginal main effect on performance. The strongest main effect was for the current-trial congruency effect and, to a lesser but still substantial degree, for stimulus repetition. The preceding-trial congruency effect was only small. Again a large interaction effect was obtained between preceding-trial and current-trial congruency, reflecting the modulation of the conflict effect by preceding-trial congruency. Participants were slower (32 ms) when the previous trial differed in congruency from the current trial (iC and cI trials).

With respect to experimental effects for the accuracy data, main effects were observed for congruency on the current and the preceding trial, and for stimulus repetition. Spatial orientation showed no main effect on accuracy. A two-way interaction was observed between the congruency of the current and of the preceding trial; again reflecting a context effect. The interaction indicative of the context effect was still present after eliminating stimulus repetition trials η^2^ = 0.23 [*F*_(1, 136)_ = 535, *p* < 0.001] for RT, and η^2^ = 0.23 [*F*_(1, 136)_ = 309, *p* < 0.001] for accuracy data).

Again, the means of the design cells from the experimental factor design were used for the analysis of individual differences in RT. The unconstrained measurement model incorporated two correlated general-speed factors for the horizontal and the vertical Simon task, respectively. This solution proved superior to a single general-speed factor (Δχ^2^ = 777, Δ*df* = 1). Additionally, nested factors were modeled for conflict adaptation, stimulus repetition, and context adaptation, each loading on its indicators regardless of spatial orientation (horizontal vs. vertical). The resulting model 2-1 had an acceptable fit (χ^2^ = 179, *df* = 79, *p* < 0.01, CFI = 0.97, SRMR = 0.03).

Starting from model 2-1, we set constraints on factor loadings in model 2-2, summarized in Table 3 The fit of this model deteriorated but deemed acceptable for the present purposes (χ^2^ = 326, *df* = 110, *p* < 0.01, CFI = 0.94, SRMR = 0.03). Strongest loadings were again observed for the general performance factors; this is also reflected in high estimations of McDonald's ω. Loadings on nested factors were smaller. As for model 1-3b the context adaptation factor had the lowest set of regression weights. However, model fit significantly deteriorated when these factors were omitted. We conclude that the conflict and the context factors capture systematic variance in people's behavior. The estimated values of ω for all nested factors (Table 4) are also indicative of coherent variance that reflects meaningful individual differences. Estimating model 2-2 with correlations between the nested factors resulted in only one significant correlation, that between the context and the conflict factors (*r* = 0.47; Δχ^2^ = 9, Δ*df* = 1). Allowing this free correlation improved the fit to χ^2^ = 317, *df* = 109, *p* < 0.01, CFI = 0.94, SRMR = 0.03.

**Table 4:** **McDonald's ω and correlations of RT from the Contrast between the Horizontal and the Vertical Simon Task with 50% Congruence Frequency**.

	**RT data**	
	**McDonald's ω from model (2–2)**	**Correlations of WM and latent factors from model (2–4)**
Vertical: general RT performance	0.983	−0.50^**^; Δχ^2^ = 19, Δ*df* = 1
Horizontal: general RT performance	0.977	−0.42^**^; Δχ^2^ = 14, Δ*df* = 1
Conflict adaptation	0.403	−0.06; Δχ^2^ = 0, Δ*df* = 1
Context adaptation	0.294	−0.04; Δχ^2^ = 0, Δ*df* = 1
Stimulus repetition	0.573	−0.10; Δχ^2^ = 0, Δ*df* = 1

Significance of correlations is indicated by ^*^ for p < 0.05 and

** for p < 0.01.

Estimating indicator means with model 2-2 led to a loss of fit leaving the model 2-3 on the edge of acceptability (χ^2^ = 533, *df* = 120, *p* < 0.01, CFI = 0.89, SRMR = 0.03). The two general performance factors had a similar factor mean, the horizontal task being faster than the vertical task by about 17 ms. The factor mean of the repetition factor was negative (mean: −18 ms) but again its variance (SD: 14 ms) implies no consistent effect for all participants. The conflict factor (mean: 33 ms, SD: 12 ms) and the context factor (mean: 27 ms, SD: 8 ms) both reflect consistent experimental effects of similar magnitude on the level of latent factors.

Testing for correlations between latent factors and WMC, WMC was introduced into model 2-2, again measured by RS, CS, and MU, with loadings of 0.79, 0.56, and 0.74, respectively, on the latent WMC factor. This model 2-4 fit reasonably well (χ^2^ = 415, *df* = 152, *p* < 0.01, CFI = 0.94, SRMR = 0.04). Correlations between WMC and the Simon task factors are reported in Table 4. The only significant correlations were observed between WMC and the general performance factors from the horizontal Simon task (*r* = −0.42, Δχ^2^ = 14, Δ*df* = 1) and the vertical Simon task (*r* = −0.50, Δχ^2^ = 19, Δ*df* = 1). Participants with higher WMC tended to perform generally faster, but they did not differ from low-WMC participants in the size of conflict and control related effects. Estimating correlations between WMC and latent factors from model 2-1 led to a better fitting model (χ^2^ = 254, *df* = 121, *p* < 0.01, CFI = 0.97, SRMR = 0.03) but did not alter any of the results reported for model 2-4.

## General discussion

This study aimed to investigate individual differences in experimental effects of conflict effects and context effects that have been taken as evidence for a general cognitive system for conflict monitoring and control. The use of confirmatory factor analyses allows isolating individual differences in experimental effects. In this way, correlations between experimental effects and other variables can be tested—something rarely done in experimental as well as individual difference research.

Descriptive results showed a consistent conflict effect for RTs as well as accuracies for all Simon tasks, that is, congruent trials were faster and more accurate than incongruent trials. The size of this effect varied considerably across participants. We also replicated the modulation of the conflict effect by the congruence of the preceding trial (i.e., the context effect or Gratton effect). Additionally, we observed effects for proportion of congruent trials and of the spatial orientation of the Simon task—although the latter was relatively weak. The mean size of the conflict effect depended on the spatial orientation. At the same time, the finding that a single conflict factor was sufficient to account for individual differences in conflict effects for both spatial orientations implies that the individual ability to deal with conflicts generalizes across the two versions of the Simon task.

The manipulation of the proportion of congruent trials affected RTs and accuracies overall, and modulated the conflict effect. Incongruent trials became faster and more accurate when they became more prevalent, whereas congruent trials became slower and less accurate compared to the baseline condition of 50% congruent trials.

Modeling individual differences was successful for RTs as well as accuracies. For the three Simon tasks of differing proportion of congruent trials (25, 50 and 75%), the three block factors represent the variance common to all RTs in each of the different experimental Simon blocks. They were highly correlated with each other, reflecting shared variance due to general efficiency in making the speeded decisions required in the Simon task, shared among all experimental conditions. The fact that we needed three different factors to represent general efficiency could be due to the fact that we counterbalanced the order of the three experimental blocks across participants. This counterbalancing scheme introduces sources of variance that are specific to individual blocks^2^. All other factors had loadings from variables in one experimental condition, defined by a single independent variable or the crossing of two independent variables. These factors represent residual variance—after extraction of the general-efficiency variance—due to the experimental manipulation. In other words, each of the more specific factors reflects individual differences in the size of one experimental effect. The independent variables caused effects on both the means and on the variance of the dependent variable, and on the covariance between different design cells. Our confirmatory factor model reflects all three effects of the experimental manipulations in the parameter estimates of factor means, factor variances, and factor loadings, respectively.

Our results offer little evidence for the hypothesis that conflict resolution is closely related to WMC and thus contradict predictions drawn from EA and load theory. In the models of RT, WMC correlated highest with the general-speed factors, which merely reflect the speed of processes common to all experimental conditions regardless of the level of conflict they incur. This result is not surprising because general cognitive speed or mental speed has repeatedly been found to be correlated with WMC (Deary, 2000; Danthiir et al., 2005; Schmiedek et al.,, 2007). From the EA theory of Kane and Engle (Kane and Engle, 2003; Kane et al., 2007) we predicted that WMC should be negatively correlated with the conflict factor, which reflects how efficiently people resolve the conflict on incongruent trials, and perhaps with the context factor, which reflects to what degree adaptations to conflict carry over to the next trial. No such correlations were observed in the models of RT or accuracy data.

The factor representing adaptations to rare incongruence allowed testing a specific prediction from EA theory regarding the frequency of congruent trials. Kane and Engle (2003) predict that the strength of conflict experienced by the cognitive system is modulated by its frequency, with conflict trials calling for more EA when they are less frequent in a block of trials. We did not observe associations between the rare incongruence factor and WMC for RT nor accuracies that would support EA theory.

Concluding, our results support the notion of systematic and psychologically meaningful individual differences in conflict and context adaptations that generalize at least across different task configurations. This supports the notion of an at least moderately general process generating these experimental effects. It is not clear, however, whether individual differences in conflict and context effects generalize across different tasks. Keye et al. (2009) did not find correlations between conflict factors of an Eriksen and a Simon task in a sufficiently large sample. Converging evidence against cross-task generality comes from an experimental study. Wendt et al. (2006) showed that the context effect in a Simon task was modulated only by preceding other Simon trials and not by trials from other experimental conflict paradigms as the Stroop or the Eriksen flanker task. The failure to find context effects with different task-sets was replicated (Akcay and Hazeltine, 2008; Rünger et al., 2010) and therefore the idea of a functional link between the context and the conflict effect by a general cognitive system ought to be questioned (see Egner, 2008, for an overview).

The lack of correlations between factors of conflict and context effects on the one hand and WMC on the other hand speaks in favor of independence between cognitive control processes and WMC. This finding is not inconsistent with conflict monitoring theory because that theory does not assign a role to WMC in conflict-related processes. Conflict-monitoring could operate as a bottom-up process that side-steps WMC (Yeung et al., 2004). The finding of no association between WMC and indicators of conflict resolution is more problematic from the perspective of EA theory and of load theory.

Our finding of independence between WMC and the efficiency of resolving response conflict in the present study, as well as in Keye et al. (2009), stands in contrast to other findings that link WMC to attentional control processes that focus the spotlight on relevant stimuli, information, or task-goals in conditions of interference (Redick and Engle, 2006; Heitz and Engle, 2007; Unsworth and Spillers, 2010). An explanation for these diverging findings could lie in differences in methodological regards. Heitz and Engle (2007) as well as Redick and Engle (2006) used an extreme-group design classifying high and low WM spans, based on only one WMC tasks (with the exception of study 3 in Heitz and Engle (2007). A latent correlational approach using several indicators for WMC is superior to extreme-group designs because of the susceptibility of the latter to misclassification and overestimation of effects (Conway et al., 2005). Assessment of WMC with a single task jeopardizes validity because individual tasks reflect a large degree of task-specific variance (Lewandowsky et al., 2010).

Unsworth and Spillers (2010) used a latent factor design to model an attentional control factor based on tasks such as the Stroop, Flanker, Antisaccade, and a psychomotor vigilance task; the attentional control factor was moderately correlated with a latent WMC factor. Their indicators of cognitive control from individual tasks, however, only displayed low correlations among each other as well as with WMC task. Thus, the data of Unsworth and Spillers (2010) are consistent with the present ones and those of Keye et al. (2009). It appears that indicators of attentional control share only a small part of their variance, questioning the notion of a unitary, general ability to control attention. Only when the small proportion of variance shared between different indicators of attentional control is extracted in a latent factor, a correlation between that variance and WMC of moderate size is detected.

### Conflict of interest statement

The authors declare that the research was conducted in the absence of any commercial or financial relationships that could be construed as a potential conflict of interest.

## Footnotes

^1^Stimulus repetition refers to the repetition of the target feature. This implies for iC or cI trial sequences a change of the irrelevant feature, e.g. for the Simon task, the same form would change its position from trial *n* − 1 to trial n.

^2^Block specific variance can have two sources, both of which are methodological. First, it could be that measures obtained in close temporal succession correlate more strongly with each other than measures obtained longer apart, for instance due to fluctuations of attention in the range of minutes and hours. Second, the counterbalancing of block order implies that a given block (e.g., the 75% block) was completed first by some participants, and last by others. Individual differences in the extent of order effects will therefore appear as variance shared by all variables from the same block but not by variables from different blocks.

## Appendix

**Table:** **Results of ANOVA for RT and Accuracy data for the horizontal Simon Tasks**.

	**RT**					**Accuracies**				
	***F***	***df*_hypo_**	***df*_err_**	***p***	η^2^	***F***	***df*_hypo_**	***df*_err_**	***p***	**η^2^**
(1) congruency on current trial	363.23	1	136	0.00	0.12	128.41	1	136	0.00	0.07
(2) congruency on preceding trial	39.42	1	136	0.00	0.00	169.12	1	136	0.00	0.03
(3) stimulus repetition	162.66	1	136	0.00	0.10	49.86	1	136	0.00	0.01
(4) congruence frequency	0.54	2	135	0.58	0.00	18.85	2	135	0.00	0.01
(1) × (2)	1116.04	1	136	0.00	0.21	564.91	1	136	0.00	0.24
(1) × (3)	3.61	1	136	0.06	0.00	8.85	1	136	0.00	0.00
(1) × (4)	212.34	2	135	0.00	0.04	96.64	2	135	0.00	0.05
(2) × (3)	165.96	1	136	0.00	0.01	39.65	1	136	0.00	0.01
(2) × (4)	2.82	2	135	0.06	0.00	85.02	2	135	0.00	0.02
(3) × (4)	4.04	2	135	0.02	0.00	4.84	2	135	0.01	0.00
(1) × (2) × (3)	28.50	1	136	0.00	0.00	1.78	1	136	0.18	0.00
(1) × (2) × (4)	16.50	2	135	0.00	0.00	39.63	2	135	0.00	0.01
(1) × (3) × (4)	0.52	2	135	0.59	0.00	0.31	2	135	0.73	0.00
(2) × (3) × (4)	16.06	2	135	0.00	0.00	9.18	2	135	0.00	0.00
(1) × (2) × (3) × (4)	4.12	2	135	0.02	0.00	14.34	2	135	0.00	0.00

**Table:** **Results of ANOVA for RT and Accuracy data for contrasting the horizontal and vertical Simon Tasks with 50% congruence frequency**.

	**RT**					**Accuracies**				
	***F***	***df*_hypo_**	***df*_err_**	***p***	**η^2^**	***F***	***df*_hypo_**	***df*_err_**	***p***	**η^2^**
(1) congruency on current trial	550.60	1	136	0.00	0.15	138.98	1	136	0.00	0.09
(2) congruency on preceding trial	50.60	1	136	0.00	0.00	114.27	1	136	0.00	0.03
(3) stimulus repetition	151.23	1	136	0.00	0.07	59.83	1	136	0.00	0.02
(4) spatial orientation	37.87	1	136	0.00	0.07	2.13	1	136	0.15	0.00
(1) × (2)	902.76	1	136	0.00	0.18	452.64	1	136	0.00	0.19
(1) × (3)	0.21	1	136	0.65	0.00	0.91	1	136	0.34	0.00
(1) × (4)	77.67	1	136	0.00	0.01	24.39	1	136	0.00	0.01
(2) × (3)	90.13	1	136	0.00	0.01	0.18	1	136	0.67	0.00
(2) × (4)	22.46	1	136	0.00	0.00	11.59	1	136	0.00	0.00
(3) × (4)	12.83	1	136	0.00	0.00	0.57	1	136	0.45	0.00
(1) × (2) × (3)	8.19	1	136	0.01	0.00	24.96	1	136	0.00	0.01
(1) × (2) × (4)	12.21	1	136	0.00	0.00	0.00	1	136	0.98	0.00
(1) × (3) × (4)	1.65	1	136	0.14	0.00	8.38	1	136	0.00	0.00
(2) × (3) × (4)	2.23	1	136	0.20	0.00	6.54	1	136	0.01	0.00
(1) × (2) × (3) × (4)	0.18	1	136	0.67	0.00	3.13	1	136	0.08	0.00

**Table:** **Unstandardized Regression Weights from Model 1-3b**.

**Indicator**		**Latent factor**	**Unstandardized regression weight**	**Standard error of regression weight**
25%_cC_id	←	RT Performance 25% congruence frequency	1.00	−
25%_cC_ni	←	RT Performance 25% congruence frequency	1.00	−
25%_iC_id	←	RT Performance 25% congruence frequency	1.00	−
25%_iC_ni	←	RT Performance 25% congruence frequency	1.00	−
25%_cI_id	←	RT Performance 25% congruence frequency	1.00	−
25%_cI_ni	←	RT Performance 25% congruence frequency	1.00	−
25%_iI_id	←	RT Performance 25% congruence frequency	1.00	−
25%_iI_ni	←	RT Performance 25% congruence frequency	1.00	−
50%_cC_id	←	RT Performance 50% congruence frequency	1.00	−
50%_cC_ni	←	RT Performance 50% congruence frequency	1.00	−
50%_iC_id	←	RT Performance 50% congruence frequency	1.00	−
50%_iC_ni	←	RT Performance 50% congruence frequency	1.00	−
50%_cI_id	←	RT Performance 50% congruence frequency	1.00	−
50%_cI_ni	←	RT Performance 50% congruence frequency	1.00	−
50%_iI_id	←	RT Performance 50% congruence frequency	1.00	−
50%_iI_ni	←	RT Performance 50% congruence frequency	1.00	−
75%_cC_id	←	RT Performance 75% congruence frequency	1.00	−
75%_cC_ni	←	RT Performance 75% congruence frequency	1.00	−
75%_iC_id	←	RT Performance 75% congruence frequency	1.00	−
75%_iC_ni	←	RT Performance 75% congruence frequency	1.00	−
75%_cI_id	←	RT Performance 75% congruence frequency	1.00	−
				−
75%_cI_ni	←	RT Performance 75% congruence frequency	1.00	−
75%_iI_id	←	RT Performance 75% congruence frequency	1.00	−
75%_iI_ni	←	RT Performance 75% congruence frequency	1.00	
25%_cI_id	←	Conflict Adaptation	1.00	−
25%_cI_ni	←	Conflict Adaptation	1.00	−
25%_iI_id	←	Conflict Adaptation	1.00	−
25%_iI_ni	←	Conflict Adaptation	1.00	−
50%_cI_id	←	Conflict Adaptation	1.00	−
50%_cI_ni	←	Conflict Adaptation	1.00	−
50%_iI_id	←	Conflict Adaptation	1.00	−
50%_iI_ni	←	Conflict Adaptation	1.00	−
75%_cI_id	←	Conflict Adaptation	1.00	−
75%_cI_ni	←	Conflict Adaptation	1.00	−
75%_iI_id	←	Conflict Adaptation	1.00	−
75%_iI_ni	←	Conflict Adaptation	1.00	−
25%_iC_id	←	Context Adaptation	1.00	−
25%_iC_ni	←	Context Adaptation	1.00	−
25%_cI_id	←	Context Adaptation	0.99	0.04
25%_cI_ni	←	Context Adaptation	0.99	0.04
50%_iC_id	←	Context Adaptation	1.00	−
50%_iC_ni	←	Context Adaptation	1.00	−
50%_cI_id	←	Context Adaptation	0.99	0.04
50%_cI_ni	←	Context Adaptation	0.99	0.04
75%_iC_id	←	Context Adaptation	1.00	−
75%_iC_ni	←	Context Adaptation	1.00	−
75%_cI_id	←	Context Adaptation	0.99	0.04
75%_cI_ni	←	Context Adaptation	0.99	0.04
25%_cC_id	←	Stimulus Repetition	0.75	0.04
25%_iC_id	←	Stimulus Repetition	0.79	0.05
25%_cI_id	←	Stimulus Repetition	0.79	0.05
25%_iI_id	←	Stimulus Repetition	0.75	0.04
50%_cC_id	←	Stimulus Repetition	1.00	−
50%_iC_id	←	Stimulus Repetition	0.90	0.05
50%_cI_id	←	Stimulus Repetition	0.90	0.05
50%_iI_id	←	Stimulus Repetition	1.00	−
75%_cC_id	←	Stimulus Repetition	0.74	0.04
75%_iC_id	←	Stimulus Repetition	0.68	0.05
75%_cI_id	←	Stimulus Repetition	0.68	0.05
75%_iI_id	←	Stimulus Repetition	0.74	0.04
75%_cI_id	←	Rare Incongruence Adaptation	1.00	−
75%_cI_ni	←	Rare Incongruence Adaptation	1.00	−
75%_iI_id	←	Rare Incongruence Adaptation	1.00	−
75%_iI_ni	←	Rare Incongruence Adaptation	1.00	−
